# Supplementary material for: Transition from natal downs to juvenile feathers: conserved regulatory switches in Neoaves
Source: Res Sq. 2023 Oct 3:rs.3.rs-3382427. Preprint. [Version 1] doi: 10.21203/rs.3.rs-3382427/v1 (PMC10602114; doi:10.21203/rs.3.rs-3382427/v1)
Supplement: Supplement 1 [file NIHPPrs3382427v1-supplement-1.pdf]

Supplementary Files

This is a list of supplementary files associated with this preprint. Click to download.

- SupplementaryFigures.docx
- SupplementaryTable1TPMtableallsamples.xlsx
- SupplementaryTable2differenceofTOGCNlevelbetweenembryonicandposthatch.xlsx
- SupplementaryTable3EnrichedReactomepathwaysforembryonicsamples.xlsx
- SupplementaryTable4EnrichedReactomepathwaysforposthatchsamples.xlsx
- SupplementaryTable5TOGCNrefoverlappedTFslevel8to10diff3.xlsx
- SupplementaryTable6TFcoexpressedgeneswithTOGCNlevel.xlsx
- SupplementaryTable7TPMmt1uniquecodingkeratinocyteAVGstringtieegg60129FT.xlsx
